# Taming widow Dimanche’s horn: excision and reconstruction of recurrent dermatofibrosarcoma protuberans of the forehead

**DOI:** 10.1093/jscr/rjaf283

**Published:** 2025-05-20

**Authors:** Surjeet Dwivedi, Nilanjan Roy, Kshitij Jyoti, Animesh Vatsa, Ayush Mathur, Pragya Sharma

**Affiliations:** Department of General Surgery, Armed Forces Medical College, Solapur - Pune Highway, Wanowrie, Pune 411040, India; Armed Forces Medical College, Solapur - Pune Highway, Wanowrie, Pune 411040, India; Department of General Surgery, Armed Forces Medical College, Solapur - Pune Highway, Wanowrie, Pune 411040, India; Department of General Surgery, Armed Forces Medical College, Solapur - Pune Highway, Wanowrie, Pune 411040, India; Department of General Surgery, Armed Forces Medical College, Solapur - Pune Highway, Wanowrie, Pune 411040, India; Department of General Surgery, Armed Forces Medical College, Solapur - Pune Highway, Wanowrie, Pune 411040, India

**Keywords:** dermatofibrosarcoma protuberans, head and neck oncosurgery, plastic and reconstructive surgery

## Abstract

Dermatofibrosarcoma protuberans (DFSP) is an uncommon soft tissue sarcoma primarily found on the trunk and proximal extremities that typically appears as a slowly progressing, firm, violet-red, or blue plaque. In this case report, we describe our experience with a patient who presented with recurrent DFSP of the forehead of size 10 × 6 cm. Patient underwent wide local excision and reconstruction with a rotational scalp flap and split skin graft (SSG) cover. No early or late complications were observed in the patient. The flaps survived completely and SSG had full uptake. This surgical technique allowed a radical excision of forehead DFSP, thus potentially decreasing tumor recurrence rate. All main reconstructive criteria, such as functional and cosmetic tissue characteristics, were completely fulfilled.

## Introduction

Dermatofibrosarcoma protuberans (DFSP) is a slow-growing, locally aggressive soft-tissue sarcoma that arises in the dermis, characterized by a low likelihood of distant metastasis but with significant potential for local recurrence affecting up to 60% of patients [1].

In this case report, we describe management of a patient who presented with recurrent DFSP of the forehead of size 10 × 6 cm. Patient underwent wide local excision and reconstruction with a rotational scalp flap and split skin graft (SSG) for complete repair of two-thirds of the forehead. It is very rare to find a recurrent DFSP of the forehead and of size as mentioned above. The proximity of the tumor to the eyes, its size, and the fact that it was a recurrent disease posed tough challenges that were addressed with solutions tailored for the patient’s needs.

## Case report

A lady in her mid-40s, with no known co-morbidities, presented with a 2-year history of a recurrent mass on the forehead. The mass was gradually progressive in size and associated with intermittent episodes of bleeding and purulent discharge for the last 2 months. Patient had a mass of around 4 × 4 cm at the same site around 3 years back when she underwent wide local excision with forehead advancement flap reconstruction at a different hospital, following which a diagnosis of DFSP was made on histopathology.

**Figure 1:**
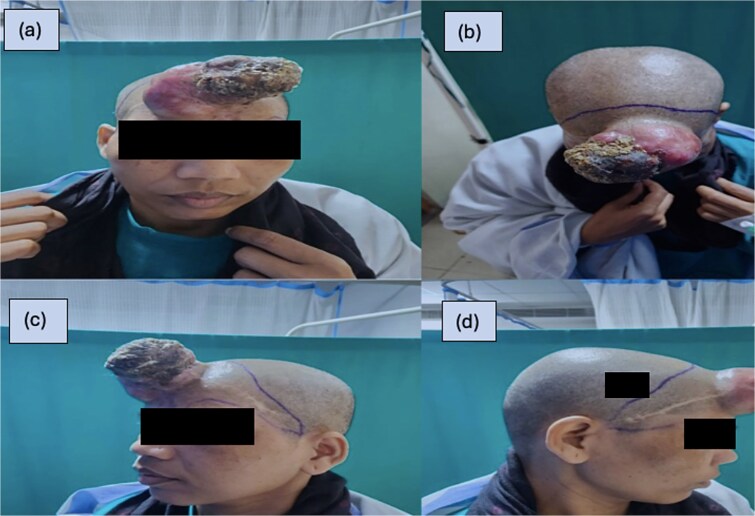
(a–d) The tumor with the marking of incision line for wide local excision.

On examination patient had a 10 × 6 cm exophytic tumor mass arising from the forehead (Fig. 1). The tumor was cylindrical in shape with well-defined margins and a rough surface. There were areas of devitalized tissue and mild oozing of blood on the tumor surface. The color of the tumor was brown with reddish areas in between. Healed skin scar of previous surgery was seen on the forehead.

Patient underwent a non contrast computed tomography head & neck which showed a 5.6 × 6 × 4.5 cm well defined heterogeneously enhancing lesion in the subcutaneous plane of frontal region with bony scalloping of adjacent frontal bone. Other routine blood investigations were within normal limits.

The patient underwent wide local excision with 3 cm margin, including the underlying outer table of skull. Reconstruction was performed with a scalp rotational flap and skin graft to cover the posterior scalp defect.

**Figure 2:**
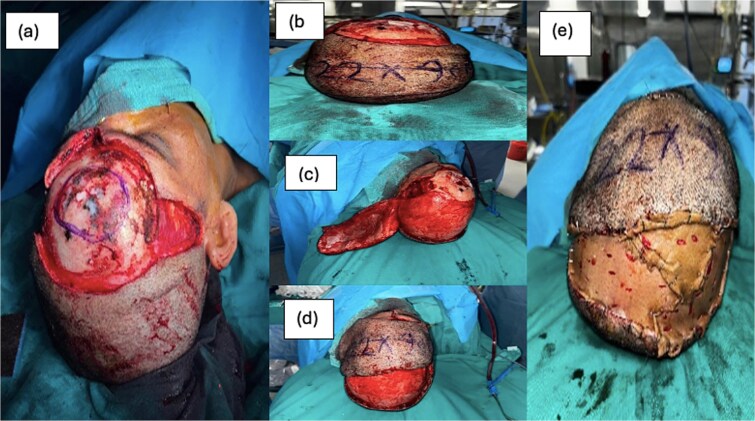
(a) Forehead defect after wide local excision, (b) marking of rotational flap, (c) raising of flap, (d) in setting of flap onto forehead defect, (e) SSG cover over posterior scalp defect.

Skin incision was marked and given from 1 cm in front of the pinna on both sides, and then continued between the two eyebrows anteriorly and 3 cm posterior to the tumor in a way to incorporate previous surgical scar. The tumor was removed en bloc with the surrounding skin margins. The outer table of the skull below the tumor was also chiseled, as the tumor appeared to be in direct contact with the periosteum intra-operatively (Fig. 2).

After finishing the resection, a 22 × 9 cm flap was marked in the scalp posterior to the defect after measuring the defect size. The scalp flap’s blood supply was provided by the left superficial temporal artery, arising from the left external carotid artery. The dissected flap was inset into the defect by rotating and sutured to the anterior margin of forehead defect. The posterior scalp defect now created was then measured, and a split skin graft was taken from left thigh and secured over it. Post operatively, there was 100% flap survival and 100% SSG uptake with no Surgical Site Infection, Patient was discharged after 10 days. Patient was followed up on outpatient department basis, and after 6 weeks, there was only 0.5 cm wound gaping over the forehead, which healed by tertiary intention.

**Figure 3:**
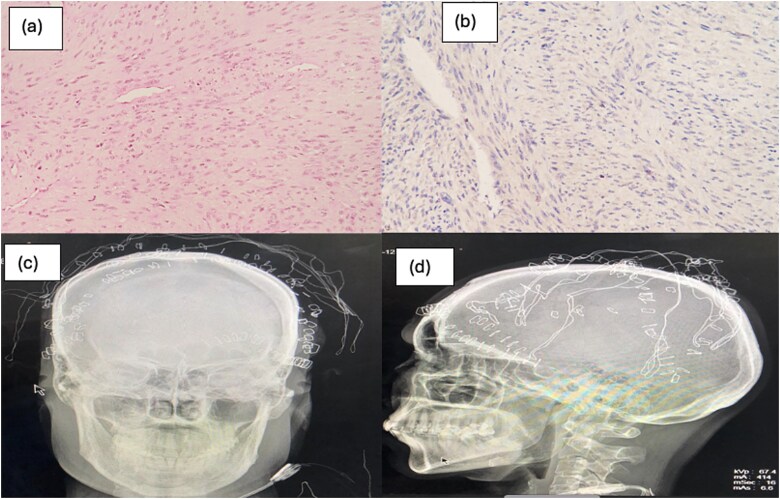
(a, b) Tumor cells arranged in fascicles and herring bone pattern and slit like and staghorn blood vessels; (c, d) post op skull X-ray anteroposterior view and lateral view, respectively, showing marking of deep posterior margin of excised tumor.

Histopathology showed an infiltrative tumor involving the dermis and subcutis. The tumor cells were arranged in fascicles and a herringbone pattern with focal entrapment of fat in the periphery. The cells had plump to ovoid vesicular nuclei, with many cells showing nucleoli. Cytoplasm was scant, with areas of mitosis and necrosis. Many slit-like and staghorn blood vessels were noted. Soft tissue posterior margin was involved; however, deep posterior margin and rest of margins were free of tumor. The outer table fragments were also free of the disease. On immunochemistry, CD34 was positive in periphery, Ki 67 was 20%–30%, SMA was positive, and p34 was of wild type (Fig. 3).

For complete recovery and no recurrence patient is being recommended for radiation therapy.

## Discussion

DFSP typically manifests as a firm, slow-growing plaque or nodule, often displaying a pink or violaceous color that may gradually increase in size. Histologically, DFSP is composed of uniformly arranged spindle cells in a storiform or honeycomb-like pattern, interwoven with fibrous tissue [2]. Recurrence is most common for tumors of the head and neck, likely because it is difficult to achieve wide margins in these areas, with reported recurrence rates as high as 50% [3]. Wide local excision remains the mainstay of treatment despite Mohs micrographic surgery gaining popularity in recent years [4]. However, no conclusive evidence has yet emerged [5].

The large defect left after resection of the lesion and previous scar posed an interesting and daunting challenge to the reconstruction team, and we used a rotation flap followed by a split-thickness skin graft to cover the donor site. Most cases reported in the literature required complex closure techniques such as mucocutaneous flaps, free flaps, and pedicle flaps with or without a skin graft [4].

**Figure 4:**
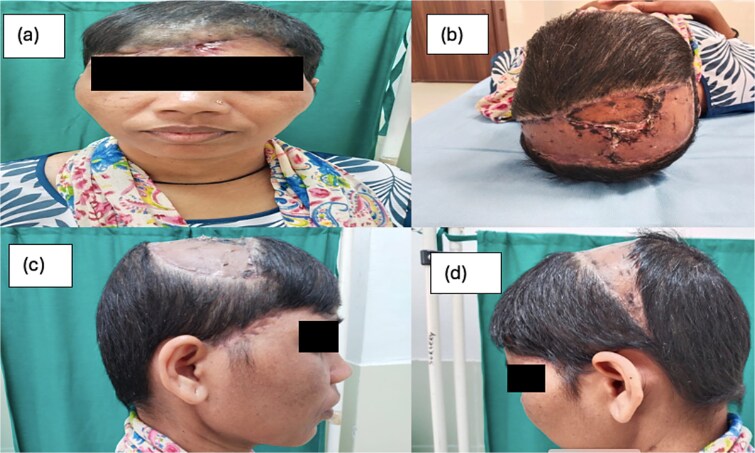
(a–d) The post op status after 6 weeks.

The role of adjuvant Radiotherapy was discussed with the patient, and considering it being a recurrent same was advised [6] (Fig. 4).

Imatinib mesylate is a tyrosine kinase inhibitor that targets platelet-derived growth factor receptors (PDGFRs). Identifying the aberrant activation of the PDGF pathway led to the hypothesis that the inhibition of PDGFR may have clinical efficacy in treating DFSP. The recent National Comprehensive Cancer Network (NCCN) guidelines recommend using imatinib for metastasis and recurrences when “disease is unresectable, or unacceptable functional or cosmetic outcomes with the resection” [6]. The location, size, and extent of involvement of the tumor provided us with a unique challenge. This case motivated us to review the important aspects of this rare tumor, besides presenting a resemblance to the cover illustration of probably the first surgical textbook we are all exposed to—Bailey and Love’s Short Practice of Surgery. This was our attempt at taming Dimanche’s horn and providing the patient relief from a stigmatizing and disfiguring disease. All surgeons must know about the frequent recurrence of this tumor, sometimes even when excised with wide margins. This surgical technique described here allowed a radical excision of the tumor, thus potentially decreasing tumor recurrence rate. All main reconstructive criteria, such as functional and cosmetic tissue characteristics, were completely fulfilled.
